# Myofibromas of the jawbones in pediatric patients. A clinicopathological study

**DOI:** 10.4317/medoral.27021

**Published:** 2025-03-23

**Authors:** Guadalupe Carolina Barajas-Torres, Norma Leticia Villanueva-Moreno, Héctor Rincón-Rodríguez, Vicente Cuairán-Ruidiaz, Horacio Márquez-González, Juan Rafael Murillo-Eliosa, Adalberto Mosqueda-Taylor

**Affiliations:** 1 Clinical Research Service. Federico Gómez Children's Hospital of México. México City, México; 2 Maxillofacial Service. Federico Gómez Children's Hospital of México. México City, México; 3 Department of Stomatology. Federico Gómez Children's Hospital of México. México City, México; 4 Clinical and experimental Pathology Service. Federico Gómez Children's Hospital of Mexico. México City, México; 5 Health Care Department. Universidad Autónoma Metropolitana Xochimilco, México City, México

## Abstract

**Background:**

Myofibromas are infrequent neoplasms that rarely occur in the jawbones. The aim of this study is to present a series of cases of these tumors affecting the jawbones in pediatric patients, as well as to describe their diagnostic methodology and therapeutic approach.

**Material and Methods:**

retrospective study of a series of myofibromas of the jawbones diagnosed and treated in a single medical institution in Mexico City from 2002-2022.

**Results:**

There were 14 cases with a median age of 6.5 years (IQR:1-12). Mandible was affected in 8 cases (57.1%), maxilla in 5 (35.8%) and only one case (7.1%) occurred in both jaws. Microscopically, the lesions were composed predominantly by spindle cells, as well as stellate and sometimes pleomorphic in shape, most of which were positive for smooth muscle actin. All cases were treated with complete excision of the lesion and only one presented recurrence.

**Conclusions:**

Once the diagnosis is confirmed and other spindle cell neoplasms have been excluded, resection with free margins represents the treatment of choice.

** Key words:**Myofibromatosis infantile, myofibroma, myofibroblast, jaw, maxilla, pediatrics.

## Introduction

Myofibroblastic lesions constitute pathological entities clinically characterized by the presence of nodular masses in muscle, soft tissues, bones, internal organs, dermis and/or subcutaneous tissue, composed predominantly of myofibroblasts, which are mesenchymal cells with characteristics of both fibroblast and smooth muscle cells (1,2). Although myofibroblasts participate in wound repair, they can also occur in various tumors, either as part of the stroma or as the main neoplastic component (3). There are different myofibroblastic lesions, which are characterized by their diverse patterns of differentiation and organization.

The concept of myofibroma was introduced in the literature in 1989 by Smith *et al*. (4), although this type of lesion had already been described in 1951 by Williams and Schrum (5) under the name of congenital fibrosarcoma. Currently, the World Health Organization uses the term myofibroma for lesions that appear sporadic and solitary and the term “myofibromatosis” for those that occur in the form of multiple lesions (1). In this way, based on the affected organs, the existence of a solitary form (single lesion) and two forms with multiple lesions have been recognized: multifocal myofibromatosis (various lesions without visceral involvement) and generalized myofibromatosis (various lesions with visceral involvement) (6), the latter with a poor prognosis when cardiopulmonary or gastrointestinal structures are involved (7). Another classification considers submucosal, intramuscular, and intraosseous types (8).

Although this condition may occur at any age, nearly 90% develop in pediatric patients, and the highest frequency has been found in men, with solitary lesions representing up to 80% of cases (6,9,10).

While it has been estimated an incidence of childhood myofibromatosis in approximately 1/400,000 (7), the true incidence of myofibromas in the oral and maxillofacial region is unknown (11). Among head and neck cases, up to 30% can affect the maxillary bones (6,12-14). Mandible is affected more frequently than the maxilla (15), and other sites of intraoral occurrence include the tongue, gingival mucosa, buccal mucosa, masseter muscle, and floor of the mouth (8,16,17).

In the head and neck, the most common clinical presentation is as an asymptomatic increase in volume (15), however, in the oral cavity it appears usually as a more or less well-defined soft tumor that generates difficulty in feeding that may produce dental displacement and/or limitation of the mouth opening (18,19).

Microscopically, it contains both single and multiple nodules that can have a biphasic cell population, composed of fascicles and/or whorls of spindle-shaped cells with abundant pale eosinophilic cytoplasm, elongated and thin nuclei with fine chromatin, which also may be arranged around thin walled blood vessels in a hemangiopericytoid-type pattern with a predominance of round to polygonal cells with a more primitive appearance, scant cytoplasm, hyperchromatic nucleus and poorly defined membrane. Mitotic activity is generally low and lacks atypia, although some lesions have a significant number of mitoses. In some cases there are areas of calcification, hyalinization and necrosis (1,3). Myofibromas are usually positive for smooth muscle actin (SMA), muscle-specific actin (MSA) and vimentin, although the frequency of labeling of the first two is variable because their expression is usually complete in mature myofibroblasts but not in all neoplastic myofibroblasts (3). Treatment for intraosseous lesions ranges from enucleation and curettage to maxillectomy and partial or total mandibulectomy (15,18).The prognosis depends on the extent and location of the lesion (20).

The purpose of this article is to present the demographic, clinicopathological features, therapeutic modalities and evolution of a series of myofibromas of the jawbones in pediatric patients treated at a single hospital in Mexico City and to compare the findings with other series previously reported in the literature.

## Material and Methods

For the development of this work, institutional approval (HIM 2021-074) was obtained from the Hospital Infantil de México Federico Gómez (HIMFG) under the declaration of the Helsinki ethical research statutes. We searched the files for all cases diagnosed as myofibroma and/or myofibromatosis with jaw involvement in patients from newborns to 18 years of age, treated by the HIMFG Maxillofacial Surgery Service in the period from 2002 to 2022.

Cases with adequate documentation and postoperative follow-up of at least 6 months were included. Those who showed incorrect histological diagnosis, inadequate records or lack follow-up were excluded. The medical records, as well as radiographs, tomography scans, histopathology reports and clinical photographs were reviewed. Data from each patient were collected in a specific format that included age, gender, location of the lesion, symptoms, time of evolution, comorbidities, imaging characteristics, clinical diagnosis, histopathology report, immunohistochemistry results, microscopic diagnosis, treatment and recurrence of the lesion. The anonymity of the patients was ensured from the generation of the database.

## Results

Seventeen cases were identified with a diagnosis of myofibroma/myofibromatosis of the jaws treated by the Maxillofacial Surgery Service of the HIMFG in the period 2002-2022, 3 of which were excluded because the records were found to be incomplete.

Table 1 shows the demographic data and clinicopathological characteristics of this group of patients. A median age of 6.5 years (IQR: 1-12) was observed, no gender predominance was observed (male: female [50%:50%]). None of the cases reported a family history of the condition, one of them had a history of mandibular trauma and another had an important comorbidity (myelodysplastic syndrome).

**Figure 1:**
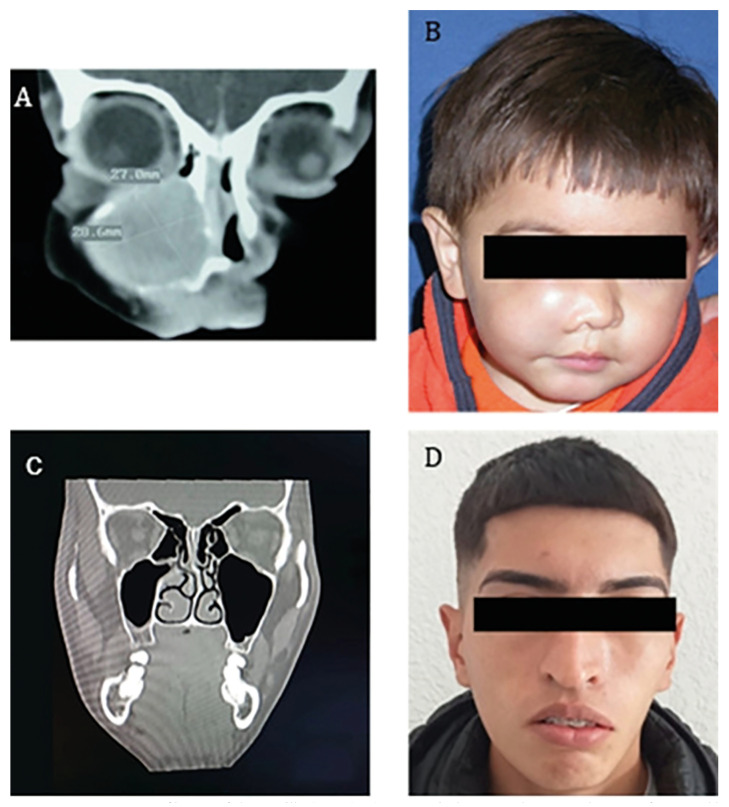
Intraosseous myofibroma of the maxilla (case 2). A) Pre-surgical computed tomography scan of 1-year-old male patient. Note the hypodensity of the lesion and cortical expansion; B) Pre-surgical facial photograph (increase volume corresponding to three months of evolution); C) Computed tomography scan after 17 years of surgical excision. D) Facial photograph after 17 years of treatment.

**Figure 2:**
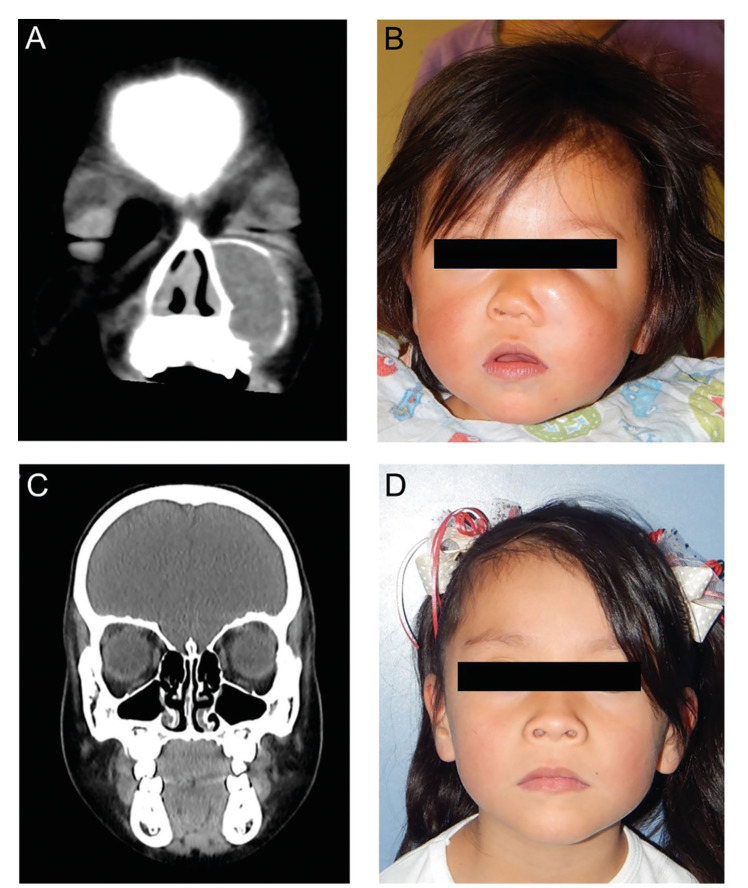
Intraosseous myofibroma of the maxilla (case 13). A) Pre-surgical computed tomography scan of 1-year-old female patient. Note the hypodensity of the lesion delimited by bone sclerosis; B) Pre-surgical facial photograph (increase volume corresponding to three months of evolution); C) Computed tomography scan after 4 years of surgical excision; D) Facial photograph after 4 years of treatment.

All lesions showed imaging features of hypodensity with cortical expansion (Fig. 1, Fig. 2), and only 5 cases presented dental displacement. The solitary intraosseous lesion type was the most common variant (92.9%).

The main reason for consultation was due to asymptomatic volume increase of a few months duration (78.6%) (Fig. 1, Fig. 2). Cortical perforation was present in 57.1% of the cases, while in 2 mandibular cases limitation of mouth opening was observed due to infiltration of the masticatory muscles. Affectation of the mandible (57.1%) was more common than that of the maxilla (35.8%), where only one of the cases corresponded exclusively to the anterior sector. Simultaneous afectation of both maxilla and mandible was found in one patient (7.1%) (Table 1).

**Figure 3:**
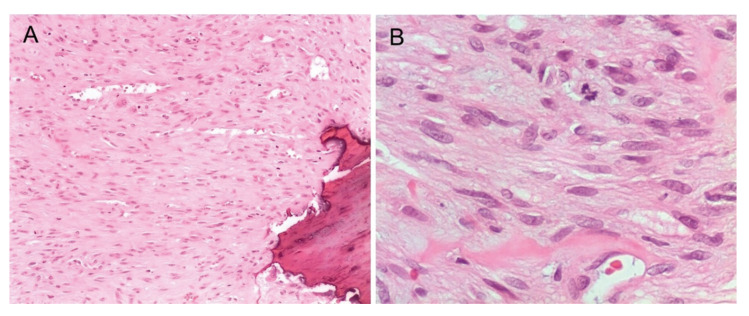
A) Photomicrograph of myofibroma showing bone destruction (100X); B) Short fascicles and mitotic figure (400X). Hematoxylin and Eosin staining.

With respect to the salient histopathological features, the presence of short or whorling fascicles of spindle or stellate cells with a myofibroblastic appearance stands out, which were characterized by having spindled vesicular nuclei, one to two nucleoli, pale eosinophilic cytoplasm and occasional normal mitotic Figures (Fig. 3). The stroma showed variable degrees (usually minimal) of collagenization or hyalinization. Some cases presented a perivascular pattern with a hemangiopericytoid appearance and most lesions were at least partly well delimited.

**Figure 4:**
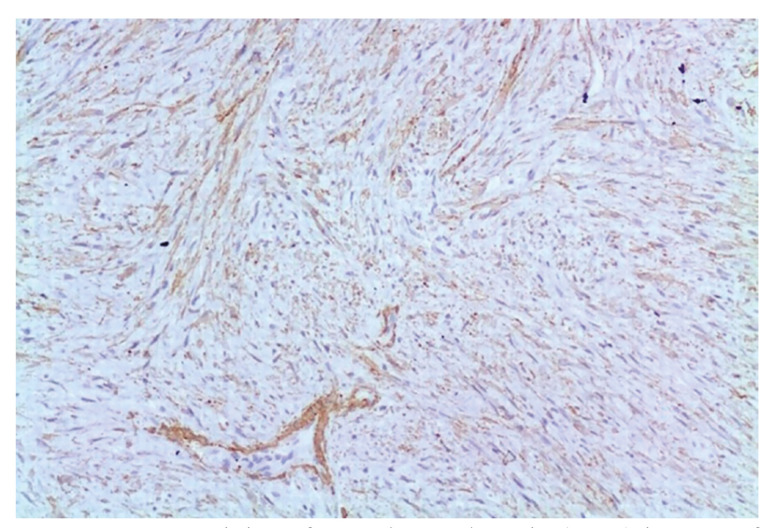
Immunostaining of smooth muscle actin (SMA) in most of tumor cells (100x).

Regarding the immunohistochemistry studies carried out in 13 out of the 14 cases included, our findings confirm that the neoplastic cells are mostly positive for SMA, MSA and vimentin (Fig. 4), without expression of other markers, such as S100 protein, H-Caldesmon or desmin, which allows us to rule out, among others, nerve sheath tumors, smooth muscle, striated muscle and fibroblastic proliferations. These findings support a myofibroblastic origin of these cells, exhibiting characteristics of intermediate cells between fibroblasts and smooth muscle cells (3).

Because most mandibular lesions presented signs of local infiltration or cortical perforation, they were treated by *en bloc*k resection and subsequent correction of the bone defect, while the largest number of maxillary cases received conservative treatment (enucleation and curettage). The mean of follow up was 6.4 years (SD 4.8) (Table 2). In most cases, the therapeutic response was favorable in the long term (Fig. 1, Fig. 2), except for case 6, who was previously diagnosed with myelodysplastic syndrome and was lost to follow-up.

## Discussion

Myofibromas are well-recognized lesions included within the spectrum of the various myofibroblastic tumor lesions that can occur in the oral and maxillofacial region, which comprises nodular fasciitis, inflammatory myofibroblastic tumor, desmoplastic fibroma and myofibroblastic sarcoma (3). According to its clinicopathological features, the differential diagnosis should include other spindle cell lesions, both benign and malignant, such as neurofibroma, leiomyoma, fibrous histiocytoma, fibrosarcoma and leiomyosarcoma (6,15,21,22).

The existing information on the clinicopathological features and management of myofibromas and myofibromatosis of the jaws comes almost exclusively from reports of isolated cases, so this study represents the largest series of cases in Mexico and provides information from the analysis of 14 patients diagnosed and treated at a single institution.

Myofibromas of the oral and maxillofacial region have been described in a wide age range (from newborns to older adults); however, it has been observed that mucosal and soft tissue lesions are somewhat more common in adults, while intra-osseous lesions are more common in the pediatric population (14,23). Series with a significant number of bone lesions (24) report a median age of 7 years, similar to the median age (6.5 years) recorded in the present study.

Due to the occurrence of this neoplasm during important stages of craniofacial development, multidisciplinary intervention becomes relevant. In addition to complete elimination of the lesion, correction of facial asymmetry, management of occlusion, rehabilitation of phoniatric sequelae, and psychological support in cases that require it should be considered.

As described in most reports (8,15), we found that the most common sign was an increase in volume of the affected bone. Unlike cases originating in the mucosa, where myofibromas tend to be slow growing and asymptomatic, intra-osseous tumors can present a rapid growth with perforation of the cortex and infiltration into neighboring structures, so they can simulate aggressive odontogenic and non-odontogenic tumors or even malignant neoplasms (15). Some authors mention the presence of symptomatology in large tumors that affect adjacent structures (20). In this work, some cases were found in which the involvement of the external pterygoid muscle caused limitation of mouth opening.

As has been mentioned in other publications (15,23), radiographic studies disclosed unilocular or multilocular hypodensity, with frequent cortical perforation. Some presumptive clinical diagnoses included odontogenic myxoma (even considering its low incidence in pediatric patients) and ameloblastic fibroma, which were ruled out by histological examination.

Among the reports of myofibromas/myofibromatosis of the oral tissues, the most commonly reported location has been the mandible (38%-66%), while the maxilla has been affected only between 5%-11% of the total number of cases (3,14). In this respect, Foss and Ellis (14) in their report of 79 cases affecting the oral and maxillofacial region observed that all lesions with intra-osseous involvement occurred in patients under 18 years of age. In our study, mandible was affected in 8 cases (57.1%), 5 occurred exclusively in the maxilla (35.8%), while one case presented involvement in both bones (7.1%). There is not an explanation for the greater involvement of the mandible reported in all series.

In a similar way to what has been reported by Allon *et al*. (23) and Abramowicz *et al*. (24), we found that although myofibromas are benign lesions, they can have aggressive behavior and produce local destruction as well as functional problems.

In our series, a maxillary lesion (case 4) occurred exclusively in the anterior area, mimicking a central giant cell granuloma, which has a predilection for this sector and should therefore be included in the differential diagnosis (25). In case 1, the mandible and maxilla were affected simultaneously, a finding that has not been found in the reviewed intraoral series (14,26).

With respect to the presence of coexisting systemic conditions, these are usually not related to the development of myofibromas, such as our case 14, which presented a dermatological condition (nevus of Ota) that have no relationship with this tumor lesion. Another patient had a diagnosis of myelodysplastic syndrome at the time of being diagnosed with mandibular myofibroma, and one year after however, this case was lost to follow-up. This is the only patient in this report where the entire jaw was affected. To our knowledge, there are no reports of maxillofacial myofibromas associated with hematological alterations, so we do not know if the underlying disease could be associated in some way with the bilateral presentation.

Some studies propose myofibroblasts as the main cells in wound repair, so the origin of myofibromas could be related to a proliferative process in response to aggression (26). Lopes *et al*. (20) reported two mandibular cases with a history of trauma at the site of involvement, in contrast to only one case present in this series.

Although a family history of myofibromatosis has been reported (27), no patient in our group had a family background of the condition.

The sudden onset, coupled with the infiltration of muscle, nerves and even glandular tissue in some cases of myofibromas of the maxillofacial region, can simulate a malignant process (15). Histopathological analysis and the support of immunohistochemistry avoid unnecessarily aggressive treatments (6,15).

Of particular interest is the provision of information in this report on 6 cases that were treated with conservative management (surgical excision), without the need for subsequent reconstruction. On the other hand, the majority of mandibular cases were treated with hemimandibulectomy followed by some reconstruction technique (only in one case was partial resection without reconstruction performed). In general, favorable results were obtained with surgical excision, similar to what has been described in other reports (16,28,29). The longest follow-up time in this series was 17 years.

In conclusion, myofibromas are rare lesions in the jawbones, their clinical presentation includes an asymptomatic increase in volume of rapid growth or have only a few months of evolution, with potential for local destruction and infiltration to adjacent tissues, which is why various locally aggressive benign and malignant lesions are included in their differential diagnosis. In addition to detailed histopathological and immunohistochemical studies, an adequate physical examination and complementary image studies are required to determine whether these are solitary or multiple lesions. Surgical resection is the treatment of choice and is usually effective in the long term, with a low recurrence rate.

## Figures and Tables

**Table 1:** Summary of demographic and clinicopathologic characteristics (n=14).

Case / Age*/ Gender	Medical history	Signs/ Symtoms	Site	Localization	Type	Evolution Time**	Immuno- staining
1/12/M	No	PLS/MI/D	Maxilla and Mandible/C	P	MM	6	ALK: - Desmin: - SMA: +
2/1/M	No	PLS	Maxilla/C	AP	S	3	Not done
3/8/F	No	PS	Mandible/C	P	S	3	ALK: - Desmin: - SMA: + Vimentin: +
4/13/F	No	PLS/D	Maxilla/C	A	S	6	ALK: + SMA: +
5/7/F	Yes^α^	PLS/D	Maxilla	AP	S	3	ALK: - Desmin: - SMA: + Ki-67: -
6/10/M	Yesᵝ	PLS	Mandible/C	P	S	4	Desmin: - EMA: - SMA: +
7/2/M	No	PLS	Mandible	P	S	3	ALK: - SMA: + Desmin: -
8/3/F	Yes^δ^	SL/MIP	Mandible	P	S	1	SMA: + ALK: -
9/6/F	No	ML/MIP	Mandible	P	S	12	ALK: - Desmin: - SMA: +
10/1/F	No	PLS/D	Mandible	AP	S	4	CD34: - PS100: not valuable Ki-67: 5% SMA: +
11/1/M	No	PLS	Mandible/C	P	S	4	ALK: - Desmin: - SMA: +
12/16/M	No	PLS	Mandible/C	P	S	6	ALK: - Desmin: - PS100: - SMA: -
13/1/F	No	PLS	Maxilla	AP	S	3	Desmin: - Myogenin: - nuclear SMA: -
14/12/M	Yes^ε^	PLS/D	Maxilla/C	P	S	5	ALK: - Desmin: - SMA: +

^α^Myofibroma recurrence (coming from another institution), ᵝMyelodysplastic syndrome, ^δ^Previous trauma, εNevus of Ota, A anterior region, AP anterior and posterior region, C cortical perforation, D tooth root displacement (lesion side) , F female, M male, MI muscle infiltration (surrounding muscles), MIP muscle infiltration (medial pterygoid), ML moderate limitation of mouth opening, MM multifocal myofibromatosis, P posterior region, PS pain swelling, PLS painless swelling, S solitary, SL severe limitation of mouth opening, *Years, **Months.

**Table 2:** Treatment and follow up (n=14).

Case	Treatment	Reconstruction	Follow up*	Recurrence
1	Mandibular en block resection and partial maxillectomy	Yes	6	NED
2	Surgical excision	None	17	NED
3	Surgical excision	None	11	NED
4	Surgical excision	None	6**	NED
5	Surgical excision	None	10	NED
6	Mandibular en block resection	None	1	ED (mandible, left area)
7	Mandibular en block resection	Yes	10	NED
8	Mandibular en block resection	Yes	12	NED
9	Mandibular en block resection	None	5	NED
10	Marginal resection	None	4	NED
11	Mandibular en block resection	Yes	3	NED
12	Mandibular en block resection	Yes	5	NED
13	Surgical excision	None	4	NED
14	Surgical excision	None	2	NED

NED No evidence of disease (myofibroma), ED evidence of disease (myofibromatosis), *years, **months.
